# Estimating variation within the genes and inferring the phylogeny of 186 sequenced diverse *Escherichia coli* genomes

**DOI:** 10.1186/1471-2164-13-577

**Published:** 2012-10-31

**Authors:** Rolf S Kaas, Carsten Friis, David W Ussery, Frank M Aarestrup

**Affiliations:** 1 DTU Food, The Technical University of Denmark, Kgs Lyngby, Denmark; 2 Department of Systems Biology, Center for Biological Sequence Analysis, The Technical University of Denmark, Kgs Lyngby, Denmark

**Keywords:** Escherichia coli, Core-genome, Pan-genome, Phylogeny, Whole genome sequencing, Genetic variation, Comparative genomics, MLST typing, Phylotyping

## Abstract

**Background:**

*Escherichia coli* exists in commensal and pathogenic forms. By measuring the variation of individual genes across more than a hundred sequenced genomes, gene variation can be studied in detail, including the number of mutations found for any given gene. This knowledge will be useful for creating better phylogenies, for determination of molecular clocks and for improved typing techniques.

**Results:**

We find 3,051 gene clusters/families present in at least 95% of the genomes and 1,702 gene clusters present in 100% of the genomes. The former 'soft core' of about 3,000 gene families is perhaps more biologically relevant, especially considering that many of these genome sequences are draft quality. The *E. coli* pan-genome for this set of isolates contains 16,373 gene clusters.

A core-gene tree, based on alignment and a pan-genome tree based on gene presence/absence, maps the relatedness of the 186 sequenced *E. coli* genomes. The core-gene tree displays high confidence and divides the *E. coli* strains into the observed MLST type clades and also separates defined phylotypes.

**Conclusion:**

The results of comparing a large and diverse *E. coli* dataset support the theory that reliable and good resolution phylogenies can be inferred from the core-genome. The results further suggest that the resolution at the isolate level may, subsequently be improved by targeting more variable genes. The use of whole genome sequencing will make it possible to eliminate, or at least reduce, the need for several typing steps used in traditional epidemiology.

## Background

The declining cost of whole genome sequencing (WGS) of bacterial pathogens has now made sequencing an option available for many scientists including those working in routine laboratories. WGS is useful in research and trend studies, but might soon be found in routine applications for diagnostics and surveillance, as well. Depending on the technology, WGS can be done in a few of hours and at low cost. Combined with the right tools, WGS makes real-time surveillance and rapid detection of outbreaks possible [1].

*Escherichia coli* is a gut commensal bacterium, as well as an important pathogen. As a commensal it acts as a beneficial member of the human microbiome in both digestion and defense against opportunistic pathogens. It is, however, also one of the most important human pathogens as it is responsible for up to 90% of all human urinary tract infections, and a frequent cause of septicemia, gastro-intestinal and other infections. *E. coli* is responsible for a large part of the more than 2 million deaths caused by diarrhea in children under the age of five in developing countries [2]. In developed countries, bacteremia is the 10^th^ most common cause of death and among the Gram-negative bacteria, *E. coli* is responsible for 30% of the cases [3]. Food borne outbreaks are also frequently observed and rapid characterization is important to detect and prevent outbreaks.

Pathogenic *E. coli* are traditionally classified on the basis of serotype and/or Multi Locus Sequence Type (MLST). Pulse field gel electrophoresis (PFGE) is also widely used, especially to detect outbreaks, because of its discriminatory power, but both PFGE and serotyping provide little phylogeneticly meaningful information. In contrast, MLST typing often lacks the discriminatory power to describe complex outbreaks [4], but can indicate some phylogenetic relationships, since it is based on the sequencing of genes, although some of these relationships might be questionable [5]. *E. coli* is also classified according to the presence of specific virulence factors in to patho-groups such as VTEC (verocytotoxin producing *Escherichia coli*), ETEC (enterotoxigenic *Escherichia coli*), EIEC (enteroinvasive *Escherichia coli*), EHEC (enterohemorrhagic *Escherichia coli*), EPEC (enteropathogenic *Escherichia coli*) and EAEC (enteroadherent *Escherichia coli*).

Apart from its role in human and animal health and diseases, *E. coli* is also an important and well-characterized model organism, which makes it one of the most sequenced organisms in GenBank, second only to *Staphylococcus aureus* in terms of the number of sequenced genomes available. This makes *E. coli* a good candidate for genome variation studies.

With the application of WGS to epidemiology, the opportunity to create better and more precise typing methods has arisen. To facilitate the future comparison of WGS data and identify clones or related strains, it is important to develop standards for classifying isolates. The genes within a genome are constantly evolving and some genes fix mutations at faster rates than others [6]. This rate is complex because it has several dependencies including gene function, selection pressure and location on the chromosome or plasmid [7].

When choosing appropriate target genes for typing purposes, it is important to know that the targets can be expected to exist in all isolates to be typed. One method for doing this is to choose genes that exist in all members of the species studied – the core-genes.

It is the aim of this study to identify core-genes and to estimate the variation within all the genes of 186 publically available *E. coli* and *Shigella* genomes from GenBank. In addition, different methods for classification of *E. coli* are evaluated. The results form a basis for future implementation of WGS as a standard typing tool for classification of *E. coli* in phylogeny and epidemiology. Standardized classification of bacteria with WGS is crucial if it is to be used in real-time surveillance and quick outbreak detection.

## Results

The Prodigal software predicted a total of 945,211 genes across all genomes. This is an average of ~5,082 genes per genome, which could be an overestimation because of the lower quality of some of the draft genome sequences. The average is ~4,837 predicted genes per genome among the complete genomes, which can be compared to the average of ~4,754 genes per genome annotated in the complete genomes in GenBank. The genes were clustered into 16,373 clusters, which represent the *E. coli* "pan-genome". The clusters were determined by MCL clustering, as described in the methods section, and are referred to as Homolog Gene Clusters (HGCs), The "soft core" is defined as all HGCs found in at least 95% of all genomes and the "strict core" is defined as all HGCs found in at least 100% of all genomes. The soft core consists of 3,051 HGCs and the strict core contains 1,702 HGCs.

**Figure 1:**
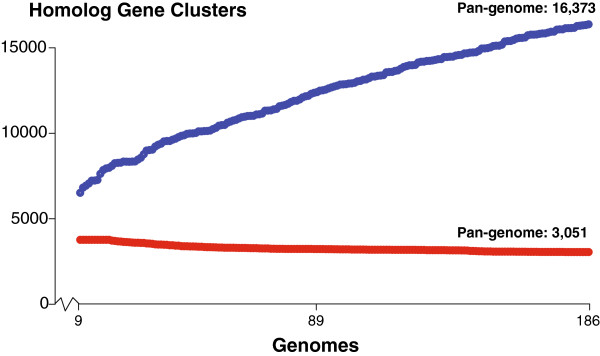
**Progress of Homolog Gene Cluster calculation as each genome is added.** Two circles exist (red & blue) for each genome added from genome no. 9 up to and including genome no. 186. Red represents the number of core HGCs after the addition of a genome and blue represents the number of pan HGCs after the addition of a genome.

The progress of the clustering algorithm is plotted in Figure 1. Each point represents the pan- and core-genome results after adding an additional genome. The x-axis starts at genome 9, because each core HGC is allowed to be missing in 9 genomes once each calculation has finished. The size of the core-genome quickly approaches 3,000 HGCs and then stabilizes. The pan-genome continues to rise with the addition of more genomes. The curve seems to become almost linear.

The first 50 added genomes are all complete genomes. There seems to be no unusual drop or rise in the core- or pan-genome, respectively, with the addition of the draft genomes.

### Variation within HGCs

**Figure 2:**
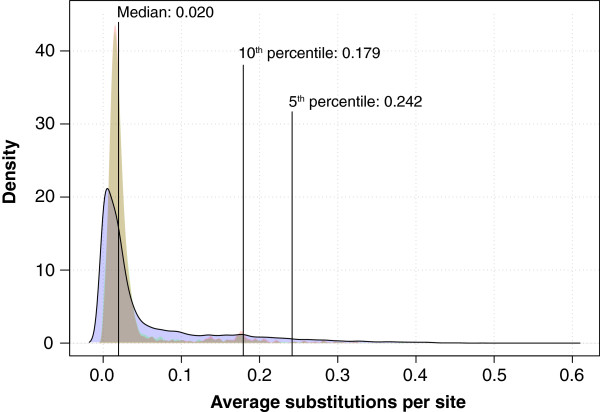
**HGC Variation plot.** A Density plot was created from the calculation of nucleotide diversity within each HGC. The blue plot was created from all the HGCs. The red plot only includes the strict core HGCs. The green plot includes the soft core (95%) HGCs. Intersection between core plots is yellow.

The distribution of variation within HGCs is shown in a density plot in Figure 2. The majority of HGCs have less than 0.020 substitutions per site. The 5^th^ and 10^th^ percentiles are also calculated. These show that 95% and 90% of the HGCs have less than 0.242 substitutions per site and 0.179 substitutions per site, respectively.

Nucleotide diversity is calculated as the average number of substitutions per site within an HGC as suggested by Nei & Li [8] (see Materials & Methods for details).

The density plot of the pan-genome (blue) has a single large top, which represents the majority of HGCs. The density plots of the soft core and the strict core are colored green and red, respectively. The intersection of the two cores is colored yellow. It can be observed that the distributions of the two core-genomes are almost identical. The tops of the core distributions are located higher on the x-axis (more diverse), than the top of the pan-genome, but the distributions are narrower, and result in lower medians (~0.018).

1,472 of the HGCs in the pan-genome have zero substitutions per site. This is mostly due to the small sizes of these HGCs; almost half of them contain only two members. One HGC contains 68 members. This HGC represents a small coding sequence of 156 base pairs. It encodes a hypothetical protein named YrhD of unknown function [Swiss-Prot:P58037, EcoGene:EG14370].

The most conserved core HGC was identical for both the soft and the strict cores. It has 188 members (substitutions per site: 0.0000467). Not surprisingly this gene cluster represents a ribosomal gene (S18).

The least conserved soft core HGC has 187 members (substitutions per site: 0.382). It represents a family of conserved genes with unknown function. The least conserved strict core HGC has 1,158 members (substitutions per site: 0.324). It represents a large cluster of ABC transporters. This large family has been reported before, and represents the diverse range of substrate specificities of the different ABC transporters, which is due to substitutions in the periplasmic binding subunit [9].

The least conserved of all the HGCs consists of 28 members (substitutions per site: 0.592). The alignment of this HGC is small and very scattered. It represents a family of transposases. The 28 members only represent 5 different genomes, 3 of which are *Shigella* genomes.

**Figure 3:**
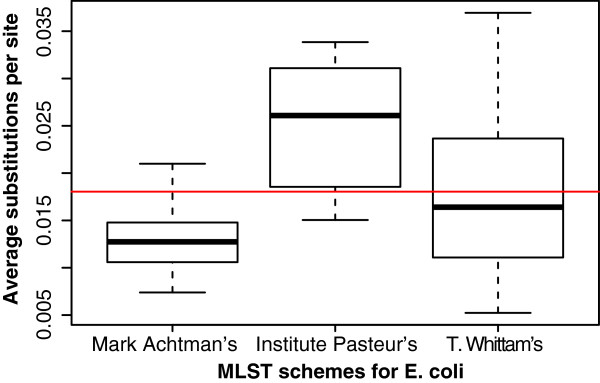
**Box plot of MLST gene variation.** A box plot presenting the distribution of nucleotide diversity within each of the three MLST schemes. The red line represents the median of percent identity for HGCs in the core (~0.018 substitutions per site).

**Figure 4:**
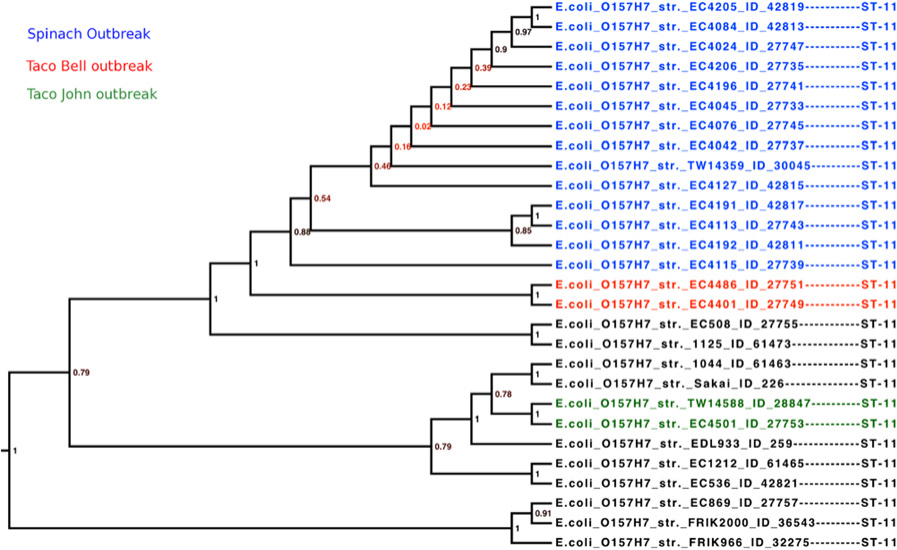
**Core-gene tree close-up on O157:H7 strains.** The tree is a close-up of the O157:H7 clade from the core-gene tree presented in Figure 6. The names has been colored according to the three outbreaks described in [21]. Blue strains represent the spinach outbreak, red strains represent the Taco Bell outbreak and the green strains represent the Taco John outbreak. Branch lengths have been modified to create the best visual output and thus have no value.

Three distinct MLST schemes exist for *E. coli*, although probably the most widely used is Mark Achtman’s set of 7 housekeeping genes (http://mlst.ucc.ie/); the Pasteur institute has created an alternative scheme, which uses 8 genes (http://www.pasteur.fr/recherche/genopole/PF8/mlst/EColi.html), and T. Whittam’s scheme uses up to 15 genes (http://www.shigatox.net/) [10-12]. A box plot for the HGCs belonging to each scheme was created and is presented in Figure 3. The genes used in each of the three MLST schemes are presented in Additional file 1. A phylogenetic tree was inferred for a selection of American outbreak isolates with ST type 11 and serotype O157:H7 using the genes from the different MLST schemes. As a proof-of-concept, a phylogenetic tree was also inferred using 7 alternative genes, which were chosen semi-randomly with a diversity ~0.03 substitutions per site. The 4 phylogenetic trees are presented in Additional file 2. None of the trees match the expected phylogeny, which can be seen in Figure 4. The tree inferred from alternative genes and T. Whittam’s scheme, seems to give the most discriminatory power.

### Distribution of functional annotations

**Figure 5:**
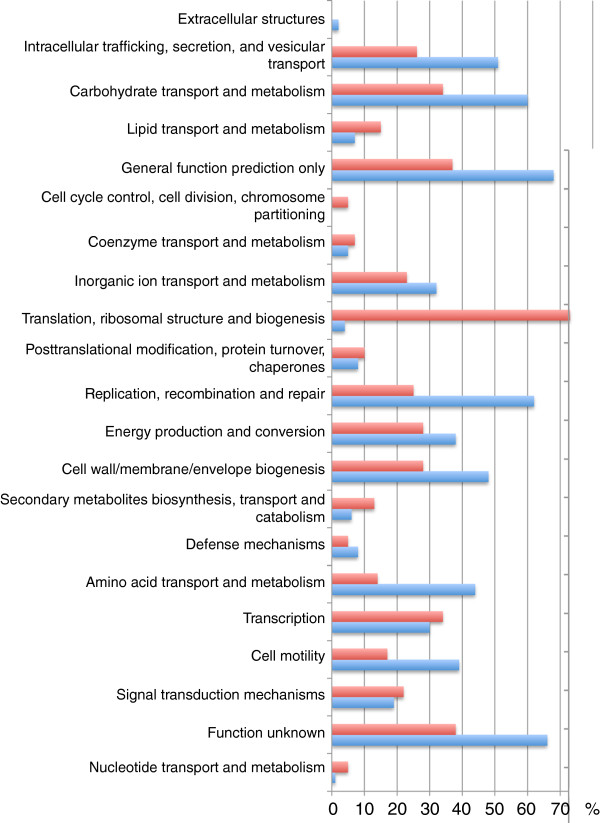
**General function of conserved and variable HGCs.** The difference in functional annotations between conserved and variable HGCs. Conserved here defined as the quarter of HGCs with the lowest nucleotide diversity (red bars) and variable defined as the quarter of HGCs with the highest nucleotide diversity (blue bars). Each HGC has a functional profile. A functional profile consists of one or more functional categories. The bars represent the percentage of HGC profiles, which contain the functional category listed to the immediate left of the bars.

All genes were annotated with functional categories, where possible, using the COG database [13,14]. The annotations for the quarter of HGCs with the highest nucleotide diversity (“Most variable genes”) and the quarter of HGCs with the lowest nucleotide diversity (“Most conserved genes”) are compared in Figure 5.

### Core-gene tree

**Figure 6:**
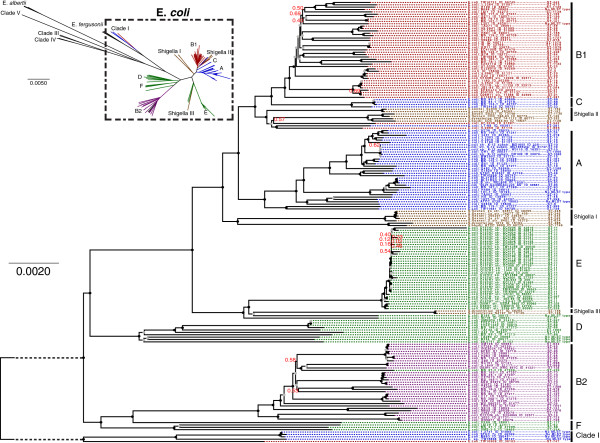
**Core-gene tree.** The *E. coli* tree was created from the alignment of 1,278 core-genes from the 186 *E. coli* genomes. MLST types are annotated to the far right of each genome name. The *Escherichia* genus tree was created from 297 core-genes. The phylotypes, as determined by the *in silico* Clermont [15] method, are marked with the colors blue (A), red (B1), purple (B2), green (D), and the *Shigella* genomes are marked with the color brown. At each node a black circle indicates a bootstrap value of 1, a grey circle a bootstrap value between 1 and 0.7 and a red number indicate an actual bootstrap value below 0.7. The dashed line in the figure represents a branch, which has been manually shortened by the authors to fit the figure on a printed page. The original tree with all bootstrap values can be seen in Additional file 2. Both trees are unrooted, but the *E. coli* tree has been visually rooted on the node leading to Clade I.

The core-gene tree of *E. coli* is presented in Figure 6. A core-gene tree of the entire *Escherichia* genus is also presented as a small inset in Figure 6. The bootstrap values are scaled from 0 to 1, and indicate the fraction of the 500 bootstrap trees that agrees with each of the nodes. Bootstrap values of 1 are replaced with a black circle and bootstrap values between 0.7 and 1 are replaced by a grey circle. The tree containing all bootstrap values can be found in Additional file 3. The four main phylotypes A, B1, B2 and D are marked by the colors blue, red, purple and green, respectively. These phylotypes were determined *in silico*, based on the work done by Clermont *et al.*[15]. Additional phylotypes, C, E, and F, have also been reported [7,16,17] and are marked with their corresponding letters in Figure 6.

In 2009 Walk *et al.*[18] reported five novel phylogenetic clades, which were phylogenetically distinct from traditional *E. coli*, but they were unable to discriminate the novel clades from *E. coli* by traditional phenotypic profiling. These are sometimes referred to as Environmental *E. coli* or the cryptic *Escherichia* lineages. In 2011 Luo *et al.* sequenced strains from four of the five novel clades [19]. The four cryptic lineages are included in the Figure 6 inset and named Clade I, III, IV, and V. Clade I is included in the *E. coli* core tree as an out-group because Clade I is very close to traditional *E. coli*. Clade I consists of 5 genomes, two of which have not, to our knowledge, been reported as Clade I strains. Using an *in silico* version of the identification procedure proposed by Clermont *et al.*[20], we further confirmed that the strains “*E. coli* STEC 7v” and “*E. coli* 1.2741” are indeed Clade I strains.

As a rule of thumb, bootstrap values above 0.7 are trustworthy, and in the core-gene tree in Figure 6, the bootstrap values are, in general, above this threshold.

Figure 4 presents a close-up of the ST 11 group of the core-gene tree. These results are in agreement with the SNP tree of a previous study on American O157:H7 outbreaks [21].

### Pan-genome tree

**Figure 7:**
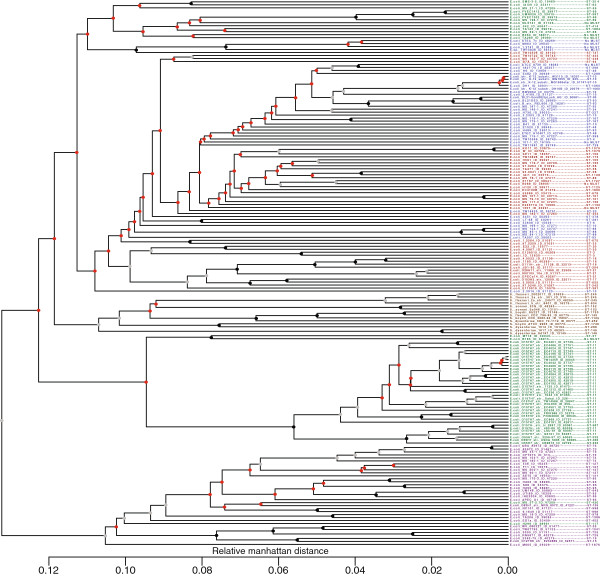
**Pan genome tree.** The tree was created based on the presence or absence of 16,373 HGCs in the 186 *E. coli* genomes. MLST types are annotated to the far right of each genome name. The phylotypes are marked with the colors blue (A), red (B1), purple (B2), green (D), and the *Shigella* genomes are marked with the color brown. Bootstrap values are annotated at each node as a percentage between 0 and 100. At each node a black circle indicates a bootstrap value of 100, a grey circle indicates a bootstrap value between 100 and 70 and a red circle indicates a bootstrap value below 70. The original tree with all bootstrap values can be seen in Additional file 3 .

The pan-genome tree is presented in Figure 7. The bootstrap values range from 0% to 100%, and indicate the percentage of the 500 bootstrap trees that agrees with each of the nodes. Bootstrap values of 100 are replaced with a black circle and bootstrap values between 70 and 100 are replaced with a grey circle. Bootstrap values below 70 are replaced with red circles. The tree containing all bootstrap values can be found in Additional file 4. The phylotypes are colored as in the core-gene tree (Figure 6).

### Validation of methods

The standard deviation of all HGCs was calculated and plotted. The Alignments of the 10 HGCs with the highest standard deviation were examined and the gene sequences were BLASTed against the nr database, Uniprot, and annotated with protein domains using InterProScan (http://www.ebi.ac.uk/Tools/pfa/iprscan/). The HGCs seem to be well defined. The HGCs were either manually annotated as virulence factors (*e.g.* adhesins) or were of unknown function. Common to these 10 HGCs is also a very large average gene size. For the HGC with greatest standard deviation (adhesin) the average genes size is ~13,000 nucleotides. See Additional file 5 for details.

Genes were annotated with functional categories using the COG database. Each gene can be annotated with several categories. In this study it will be referred to as the “functional profile'” of the gene. Ignoring the functional profile “unknown function”, 4,123 HGCs contained genes with an identical profile. 12,189 HGCs could not be annotated. 59 HGCs contained genes with two different profiles, and 2 HGCs contained genes with more than two profiles. These two HGCs were examined and seem to be well defined. The 4,123 HGCs annotated with a single profile represents ~75% of all the genes.

In this study we include both draft and completed genomes. To estimate whether or not inclusion of draft sequences influences nucleotide diversity, we tested three datasets. One consisted of the 50 complete genomes, the other two consisted of 50 draft genomes randomly picked (without replacement). Clustering and nucleotide diversity calculation for all three datasets were performed. The two pan-genomes of the draft sequences seemed to be slightly higher than for the complete one. Virtually no difference in the distribution of nucleotide diversity was observed. See Additional file 6.

## Discussion

In this study we identified core-genes and estimated the genetic variation among 186 publically available *E. coli* and *Shigella* genomes. Here, we will have a brief look at how *E. coli* is currently classified, how it fits our data, and discuss how these results may form a basis for future implementation of WGS as a standard typing tool for classification of *E. coli* in phylogeny and epidemiology and understanding *E. coli* evolution.

The dataset analyzed was obtained from GenBank and is publically available from NCBI. Two data quality issues are immediately encountered when using sequence data produced by others and from several different researchers: genome annotation and sequence quality. The annotation of the sequences can be very different, due to different annotation pipelines. Some annotations are manually curated and others are not. The completeness of each sequence can vary – some completed sequences are more “complete” than others. Chain *et al.* suggested a list of 6 categories in which all sequenced genomes could be defined based on their level of completeness [22]. In an attempt to overcome the bias from different annotations all genomes were annotated using the Prodigal gene finder [23] which provided consistency across the entire data set.

Sequence quality is also a concern. Unfortunately there hasn’t been much focus on the issue, and publications estimating error rates in sequence databases are scarce. To our knowledge there are no recent publications estimating error rates in bacterial genomes deposited in GenBank. Wesche *et al.* estimated error rates in the mouse DNA sequences deposited to GenBank in 2004 [24]. They found an error rate of 0.1% in coding DNA sequences. This is lower than the estimate done in 1988 for all GenBank sequences deposited at the time, which demonstrated an error of ~0.3% [25].

Eukaryotes in general have much more complex genomes, due to introns, exons and complex repeats, which in turn leads to a higher than expected error rate. Sequencing technologies and assembly have also improved significantly since 1988. It is hypothesized that a conservative estimate of sequence errors in bacterial sequences deposited to GenBank today is less than 0.1%. Consequently an average *E. coli* gene (~1000bp) will contain approximately 1 error per gene.

Most errors caused by NGS technologies comes from insertions and deletions (indels), which will be completely ignored, due to the way nucleotide diversity is calculated. Therefore the errors, which are actually having an effect on the nucleotide diversity calculations, are probably lower than 0.1%. Because of these facts, it is believed that errors will, at most, cause 0.001 additional diversity to any of the variation calculations, and we believe that this is probably a very conservative estimate.

Sequencing errors, both indels and nucleotide changes can, however, cause genes to be truncated. Touchon *et al.* showed that at least 23 essential housekeeping genes were missing in their core-genome [7], and genomes missing these genes turned out to contain truncated versions of the “missing” genes. It was hypothesized that this was probably due to sequencing errors. Owing to the possibility of sequencing errors accidently “deleting” genes from a genome, we also present the results for the soft core in this study.

Another issue, which sets a limit on our ability to interpret the results, is the lack of metadata, or specifically, the lack of a method for obtaining relevant metadata in an automated way. The amount of sequence data available now makes it unfeasible to email the corresponding author for each available genome to obtain its metadata. The community is aware of the increasing need for metadata and The Genomics Standards Consortium has suggested the Minimum Information about a Genome Sequence (MIGS), some of which is being incorporated into more recent GenBank files [26].

### Pan- and core-genome

The core-genomes of *E. coli* and *Shigella* have been estimated in several studies. Lukjancenko *et al.* estimated the core-genome in 2010, from 61 genomes, using a single linkage clustering method and found it to be 1,472 HGCs if only *E. coli* was considered [5]. Vieira *et al.* estimated the core-genome in 2010 from 29 *E. coli* and *Shigella* genomes using the orthoMCL algorithm and found the core-genome to consist of 1,957 gene clusters [27]. In 2004 Fukiya *et al.* examined the core-genome from 22 *E. coli* strains using comparative genomic hybridization and estimated it to consist of approximately 2,800 shared open reading frames among all the strains [28]. Willenbrock *et al.* used high-density micro arrays to estimate the core-genome of 32 *E. coli* and *Shigella* genomes, and estimated the core-genome to be around 1,563 genes [29]. Chattopadhyaya *et al.* estimated the core-genome to consist of 1,513 genes among the 14 *E. coli* strains considered in their study [30]. Touchon *et al.* estimated the core-genome in 20 *E. coli* to be 1,976 genes and the pan-genome to consist of 11,432 genes. Thus, in previous studies (with fewer genomes) the size of the core-genome seems to fluctuate between 1,000 and 3,000 genes and generally conforms to the expectation that the core-genome would decrease, as an increased number of strains are analyzed, which might be an artifact of truncated genes due to sequencing errors.

In this study we found the soft core-genome to consist of 3,051 HGCs (Figure 1) for 186 genomes. In contrast to previous studies, we allowed a soft core-gene to be missing in up to 5% of all the genomes. If the strict core (HGC must be found in all genomes) was considered, the core-genome shrinks to 1,702 HGCs. It fits well within previous estimations made with the same strict cutoff.

The pan-genome has also been estimated in many studies and will probably continue to increase as more genomes are sequenced. In one study, the pan-genome of *E. coli* has been estimated to be as large as 45,000 gene families [31]. Another study suggests that the bacterial pan-genome is infinite [9]. Additional *E. coli* isolates, including some more distinctly related to those already sequenced, should be sequenced to obtain a more complete picture of the *E. coli* pan-genome.

### Gene variation

The joint core-genome diversity plotted in Figure 2 (yellow) has one large top, which suggests that for most core-genes there is little room for diversity. Several smaller tops are also observed. We examined some HGCs that are part of the larger of the smaller tops (~0.17 substitutions per site). In both cases the HGC consisted of a gene coding for an enzyme and its isozyme counterpart. As for the case of one of the most diverse core families, the ABC transporters, the high diversity is due to different genes coding for proteins having very similar functions.

The pan-genome diversity plotted in Figure 2 has one large top and the distribution is much broader, as would be expected, due to the inclusion of the accessory genes.

No single, officially recognized system for classification of prokaryotes exists at the present time. The “polyphasic approach” is the most popular, and includes phenotypic, chemotaxonomic and genotypic data [32]. As for the genotypic data, this means that two genomes have to be 70% similar in order to be considered the same species. It has been shown that >70% similarity corresponds to an average nucleotide identity among the core-genes of >95% [32]. These results are supported by the median ~0.018 substitutions per site for the joint core found in this study.

Figure 3 shows that the genes from the Mark Achtman MLST scheme and the T. Whittam MLST scheme, in general, have less diversity than the majority of core HGCs. This is a bit surprising because the more variation in a gene, the greater the potential to be able to distinguish different strains.

The Pasteur MLST scheme seems to contain quite diverse core-genes, but also contains some which are more conserved than the average core-genes. This raises the question of whether or not a selection of more variable core-genes could be made, which, in turn, could provide higher resolution. Variability is, of course, not the only consideration when choosing MLST genes, *e.g.* an MLST scheme should not contain genes that are candidates for horizontal gene transfer, they should not be paralogous, and they should reflect the true phylogeny as much as possible. It is beyond the scope of this study to present a new MLST scheme, but it will be demonstrated how resolution could improve by choosing more diverse MLST genes. 7 core HGCs were chosen semi-randomly, with variation around ~0.03 substitutions per site. Genes were chosen with variation higher than average, although not so high as to include paralogous genes. We found the corresponding genes in a set of 24 O157:H7 strains, aligned them and built a phylogenetic tree. Phylogenies were also inferred using each of the other three MLST schemes (see Additional file 2). We compared the MLST phylogenies with a published SNP tree created from these strains [21]. There is almost no variation found in the traditional Mark Achtman MLST scheme genes in these strains. In the alternate MLST scheme tree there is more variation and in turn more resolution. T. Whittam’s scheme has the best overall resolution, probably due to the fact that T. Whittam’s scheme contains twice as many genes as the other MLST schemes. None of the MLST phylogenies presents the expected topology. It seems unlikely that any selection of genes this small will ever be able to infer a robust phylogeny for an *E. coli* outbreak. At this point in time, there is probably no need to chase after a better MLST scheme, as WGS will probably make MLST typing obsolete with time. For most scientists, WGS is already less expensive than MLST typing [33]. WGS is, in general, far more promising, since it enables the use of entire core-genomes and SNPs (see core-gene tree discussion).

Barrick *et al.*[34] documented the mutations fixed in a specific *E. coli* strain over 40,000 generations *in vitro*. We looked at the genes and their corresponding HGCs in which these mutations occurred, but found no significant trend with regard to the variability of the mutated genes (data not shown).

### Gene function distribution

Most HGCs could not be annotated with a functional category (~12,000); this corresponds to ~25% of all the genes.

The annotations of the HGCs are presented in Figure 5. As expected, the conserved genes are overrepresented in the “ribosomal” category, and even though there are only a few HGCs found in the “extracellular” category, they are exclusively from the variable HGC pool.

### Core-gene tree

*E. coli* as a species contains within it a large diversity of adaptive paths. This is the result of a highly dynamic genome, with a constant and frequent flux of insertions and deletions [7,16]. Touchon *et al.* shows that the dynamic genome is compatible with a clonal population structure such as *E. coli*, since most gene acquisitions and losses happen in the exact same locations (“hotspots”). Hence the phylogenetic signal is still strong within the core genome even though recombination and lateral gene transfer is frequent [7].

The concatenated gene tree in Figure 6 demonstrates this strong phylogenetic signal quite well by the high fraction of confident nodes (confident nodes having a bootstrap value above 0.7). The tree also agrees with the MLST types. None of MLST types are actually split with the exception of ST-10, ST-11 and ST-93. In the ST-93 clade there is a single strain, which could not be typed by the *in silico* MLST algorithm. It is the draft genome of *E. coli* 101–1. Perfect matches for all 7 alleles are found, for the MLST scheme, but the combination is unknown. Its location within the ST-93 clade is valid though, since the unknown type is due to a single locus change (fumC-11 --> fumC-130). *E. coli* H 2687 with ST-587 is also a single locus variant of ST-11. ST-10 is split by ST-1060 and ST-167. Since the two strains of ST-1060 are sub-strains of K12, which is classified as ST-10, these fit inside the ST-10 clade. ST-167 is a single locus variant of ST-10.

All phylogroups (A, B1, B2, C, D, E, and F) also correspond very well with the core-gene tree. Only a few strains seem to violate the groups. *E. coli* MS 57 2 is classified as D, but the tree strongly suggests that it should belong to the B2 group. Gordon *et al.* showed that using the Clermont PCR multiplex method could lead to erroneous classification of phylotypes [35], in particular, classifying B2 phylotypes as D phylotypes were shown to be frequent. They proposed a new gene target, “ibeA”, which will distinguish most B2 types from D types. *E. coli* MS 57 2 contains the gene target ibeA, which confirms its placement within the B2 phylogroup [35].

The tree supports the claim that B2 and F are the ancestral groups followed by D and then the sister groups B1 and A [7,16,36].

The fact that phylotyping and MLST typing fit so nicely with the core-gene tree, both confirms the highly clonal nature of *E. coli* and supports the use of core-genes to infer the “true” *E. coli* phylogeny.

To obtain a resolution high enough to be used in short term epidemiology, researchers have turned to inferring phylogenies from Single Nucleotide Polymorphism (SNP). SNP trees have, with much success, been used previously to describe complex outbreaks in detail [4,37]. However, to create a SNP tree, a good reference is needed and it is also frequently necessary to sort out false SNPs. The latter will always be subject to some controversy, because determination of a false SNP call will seldom be a completely objective call.

The creation of a core-gene tree requires no subjective alterations, which, in turn, also makes them much easier to automate and replicate than SNP trees. Figure 4 presents the E clade of the core-gene tree, and demonstrates the ability to differentiate three American *E. coli* O157:H7 outbreaks from each other. This is slightly better even, than the SNP tree published by Eppinger et. al [21].

In a case where the core-gene tree does not provide enough resolution, better resolution might be obtained by focusing on the more variable genes; in these cases care should be taken not to focus on paralogous to infer phylogeny. Whether this is possible is doubtful, and will require further studies with strains of known origin and relationship for validation.

Based on many various typing methods, *Shigella* consistently has been shown to belong within the *E. coli* species [5]. Indeed, within Figure 6, all *Shigella* species can be seen to fall within the *E. coli* clade. How *Shigella* got the ‘shiga toxin’ and other pathogenicity genes has two opposing theories. One theory suggests that all the “*Shigella* genes” originated from one ancestral plasmid [38]. Another theory suggests that *Shigella* originated from three different *E. coli* species, which, independently of each other, acquired the “*Shigella* genes” [39]. Our core-gene tree (Figure 6) supports the latter theory, which is not surprising, since the theory was based on trees created from housekeeping genes. The core-gene tree fails to group the *Shigella* species. *Shigella* are classified based on their virulence factors, which are probably poor phylogenetic targets, and thus does not explain the “true” relationship between the *Shigella* species.

### Pan-genome tree

The pan-genome tree is based on the absence or presence of all the HGCs of the pan-genome. It has been reported by Touchon *et al.* that gene conversion events are more likely than point mutations in *E. coli*. From this they conclude that the contribution made by recombination events outweigh site-level mutations as an evolutionary mechanism [7].

The pan-genome tree differs from the core-gene tree, because it is focused on those genes that are absent between the genomes. Since all the core-genes will be present in all genomes these will not in any way influence the phylogenetic relationship in this tree.

The pan-genome tree does not have as confident nodes as the core-gene tree. The deeper nodes are almost all below 50%. However, the nodes close to the leaves are quite confident and a majority of these reaches 70-100%.

These results are in agreement with the previously mentioned study by Touchon *et al.* The gene diversity in *E. coli* creates a poor phylogenetic signal between distantly related strains, since the signal is only made up from very few fixed ancestral insertions. This is due to the high gene flux in *E. coli* which causes only closely related strains to share a significant amount of accessory genes [7].

There are many similarities between the core-gene tree and the pan-genome tree, but also some obvious differences. The pan-genome tree does not divide the strains as nicely into the different phylogroups as the core-gene tree. The MLST type clades are also more divided than is the case for the core-gene tree. These results might not be that surprising, since both phylogroups and MLST types are based on a small set of core-genes and the pan-genome tree actually ignores these genes.

The pan-genome tree, due to one single *Shigella* clade, supports the “one origin” theory, as opposed to the core-gene tree, which supports the “three origins” theory of *Shigella*. Since the definition of *Shigella* is based upon a group of genes which gives it its pathogenic characteristics, it makes perfect sense that the pan-genome tree, which focuses on gene presence/absence, is able to isolate the *Shigella* genus into one single clade.

This convergence for *Shigella* has been observed previously by calculating the “metabolic distance” between *E. coli* strains. Vieira *et al.* suggests that this inconsistency between genetic distance and metabolic distance is proof that the *Shigella* metabolic networks have evolved quickly by genetic drift [27].

Both trees fail to divide the *Shigella* genus into any species clades, which further supports the argument that the taxonomy within *Shigella* might not be optimal.

### Future perspectives

The core-gene tree in this study had a surprising capability to differentiate between closely related outbreak strains. However, more resolution might be needed to infer phylogenies or detect short-term outbreaks. In these cases, it might prove useful to put more weight on the variable regions of the genome. Further studies are needed to decide if this is a meaningful approach.

The results found in this study may lay ground for further studies into how we might create a standardized method for defining *E. coli* strains. To do this, studies are needed in which *E. coli* strains from different outbreaks and with different degrees of relatedness are sequenced and compared. Although “Single Nucleotide Polymorphism” (SNP) analysis was not done in this study, SNP potentially could be a powerful typing technique and will need to be included in future studies. This will, however, make more sense with a dataset that has been selected for this purpose.

It is becoming more and more apparent that a global epidemiological detection system is important, and for a global collaboration to be successful, standards are crucial.

## Conclusions

Genes across different *E. coli* genomes are, in general, very well conserved. A pan-genome of 16,373 HGCs was found. A soft core-genome of 3,051 HGCs was found using a 95% cutoff, meaning that each HGC had to be found in 95% of the genomes to be considered a “core” HGC. With no genomes lacking HGC, we reached a core genome of 1,702 HGCs.

A pan-genome tree was created based on the absence or presence of genes. This method demonstrated the convergence of the *Shigella* lifestyle.

A core-gene tree was created based on the concatenated alignments of the core-genes. The core-gene tree was able to classify MLST types and phylotypes. We found that most genes used for MLST typing are less diverse than the majority of core-genes.

The core-gene tree showed a surprising capability of distinguishing a set of O157:H7 outbreak strains, and even seemed to do better than a SNP tree [21] created from the same strains. Future studies into a global standard for *E. coli* typing, should include a core-gene tree method, possibly combined with resolution improvement by focusing on variable genome regions, the latter is doubtful and remains to be tested.

The use of WGS will make it possible to eliminate, or at least reduce, the need for several typing steps used in traditional epidemiology. We are convinced that WGS is the optimal way forward in studying the phylogeny and epidemiology of *E. coli*.

## Methods

All genomes analyzed were downloaded from GenBank at the National Center for Biotechnology Information (NCBI - http://www.ncbi.nlm.nih.gov/) on the 18^th^ of April 2011. All draft and complete genomes were downloaded; a few were excluded due to content and quality. Draft genomes with fewer than 104,000 base pairs, and/or in more than 1,000 contigs were excluded. “*Shigella* sp. D9” with Genbank project ID 32507 was also excluded due to some very odd behavior in our analysis. We ended up with 171 *E. coli* and 15 *Shigella* genomes. The list of the 186 genomes can be found in Additional file 7. For each genome we predicted tRNAs with tRNAscan-SE version 1.23 [40] and rRNAs using rnammer [41] while gene prediction (excluding partial genes) was done using Prodigal version 2.6 [23]; *in silico* phylotyping was performed using in-house software, based on the presence or absence, determined by BLAST [42], of the two genes *chuA*, and *yjaA*, as well as the segment TspE4.C2 (unpublished), as proposed by Clermont *et al.*[15], and the MLST typing *in silico* was done using the MLST predictor at http://www.genomicepidemiology.org/[33]. The same set of tools was also used for all the annotated genomes in GenBank in order to obtain consistency in the gene comparisons. The differences between the annotations made in this study and the annotated genomes are listed in Additional file 7.

### Homolog gene clusters (HGCs)

Genes with similar sequences are likely to have similar functions and homologous gene clusters (HGCs) are generated by sequence similarity. In the ideal case, all occurrences of a specific gene from all the genomes will cluster exclusively into the same HGC. Using BLAT [43] all genes from all genomes were aligned against each other. The settings for BLAT were set to an E-value of at least 10^-5^. The MCL software, based on the Markov Clustering Algorithm, developed by van Dongen [44] was then used to create the HGCs from the BLAT alignments.

This clustering approach has previously been applied to both Campylobacter [45] and *E. coli*[27]. The MCL software also does the clustering in orthoMCL software/web-service [46] (orthomcl.org).

### Estimation of variation within HGCs

Multiple alignments were made for all HGCs using MUSCLE version 3.8.31 [47]. The multiple alignments were then used as input to VariScan version 2.0 [48], which calculated the nucleotide diversity based on the method suggested by Nei & Li [49]. At the gaps in the alignments, at least 10% of the members (or at least 2) had to have non-gap characters in the gap position to be included in the diversity calculation of the alignment. The “member cut-off” parameter was also set to 50% and 90%, we detected virtually no difference in the diversity distributions (data not shown).

### Core- and pan-genome

The core- and pan-genomes were defined by HGCs. The soft core-genome was defined as all HGCs that had members in at least 95% of the 186 genomes, equivalent to at least 177 genomes of the 186 genomes. The strict core-genome was defined as all HGCs that have members in all genomes. The pan-genome was defined as all HGCs.

### Functional annotation

All genes were blasted against the COG database [13], hits with an E-value > 10^-5^ were considered significant; only the best hits (highest bit score) were extracted. The functional profile of the best hit was then assigned to the query gene.

HGCs were annotated with the functional profile, which was dominant between the members of the HGC. This also included “not in COG”.

### Core-gene tree

A core-gene tree was created for all the members of the *Escherichia* genus and another one was made for only *E. coli* and *Shigella*. Both are presented in Figure 6.

To create a core-gene tree, all genes not found in all genomes were removed. A multiple alignment for each gene was then done using MUSCLE version 3.8.31 [47]. The alignments were then concatenated. 500 resamples of the alignment were created with Seqboot version 3.67 [50]. Distance matrices were calculated for the initial alignment as well as for each of the 500 resamples using dnadist version 3.67 [50]. Trees were then created using FastME from NCBI [51] and the tree from the original alignment was compared to the 500 trees from the resamples using CompareToBootstrap [52].

FigTree (http://tree.bio.ed.ac.uk/software/figtree/) has been used to visualize the final core-gene tree. The tree is unrooted, but has been visually rerooted with FigTree on the node leading to Clade I.

### Pan-genome tree

A phylogenetic tree was created based upon the absence or presence of all HGCs and a hierarchical clustering based on calculations of the Manhattan distance between each HGC. Singletons were ignored. The tree was created with the R package, as previously described by Snipen & Ussery [53].

## Competing interests

The authors declare that they have no competing interests.

## Authors' contributions

RSK carried out comparative genomics analysis, did interpretation of results and drafted the manuscript. CF helped do the comparative genomics analysis, did interpretation of the results and helped to draft the manuscript. DWU and FMA helped with interpretation of the results and to draft the manuscript. All authors were involved in conception and design. All authors have read and approved the manuscript.

## Supplementary Material

Additional file 1 **Genes used in MLST schemes.** Lists of the three groups of genes used in the Mark Achtman, Pasteur institute, and T. Whittam MLST schemes. Click here for file

Additional file 2 **MLST phylogenies of O157:H7.** Four phylogenetic trees inferred from four different MLST schemes. Tree A is inferred from Mark Achtman’s MLST scheme, tree B is inferred from the Pasteur MLST scheme, tree C is inferred from T. Whittam’s MLST scheme and tree D is inferred from the alternative MLST scheme used in this proof of concept case. Click here for file

Additional file 3 **Core tree with all bootstrap values.** The tree was created from the alignment of each of the 1,278 core genes from the 186 *E. coli* genomes. MLST types are annotated to the far right of each genome name. The phylotypes are marked with the colors blue (A), red (B1), purple (B2), green (D), and the *Shigella* genomes are marked with the color brown. Click here for file

Additional file 4 **Pan-genome tree with all bootstrap values.** The tree was created based on the presence or absence of 16,373 HGCs in the 186 *E. coli* genomes. MLST types are annotated to the far right of each genome name. The phylotypes are marked with the colors blue (A), red (B1), purple (B2), green (D), and the *Shigella* genomes are marked with the color brown. Bootstrap values are annotated at each node as a percentage between 0 and 100. Click here for file

Additional file 5 **Annotation of highly deviating HGCs.** Manual annotation of the 10 HGCs with the highest standard deviation in gene size. The annotation is based on blasting the gene members against the nr database, Uniprot and running the sequences through InterProtScan. Click here for file

Additional file 6 **Complete versus draft nucleotide diversity distributions.** The nucleotide diversity distribution is plotted for both the core-HGCs and the pan-HGCs of the three datasets: complete (red), draft1 (blue), and draft2 (green). Click here for file

Additional file 7 **Table of complete dataset. The table shows the dataset used for the article.** The “GB genes” column indicates the number of genes annotated in the corresponding GenBank file. The “Prod genes” column indicates the number of genes that was found with prodigal for this study. Click here for file
