# Supplementary material for: Estimating variation within the genes and inferring the phylogeny of 186 sequenced diverse Escherichia coli genomes
Source: BMC Genomics. 2012 Oct 31;13:577. doi: 10.1186/1471-2164-13-577 (PMC3575317; doi:10.1186/1471-2164-13-577)
Supplement: Additional file 1 — Genes used in MLST schemes. Lists of the three groups of genes used in the Mark Achtman, Pasteur institute, and T. Whittam MLST schemes. [file 1471-2164-13-577-S1.pdf]

Mark Achtman's MLST scheme:
adk
fumC
gyrB
icd
mdh
purA
recA

Institute Pasteur's MLST scheme:
dinB
icd
pabB
polB
putP
trpA
trpB
uidA

T. Whittam's MLST scheme:
arcA
aroE
aspC
clpX
cyaA
dnaG
fadD
grpE
icdA
lysP
mdh
mtlD
mutS
rpoS
uidA
