# Supplementary figures and images for: Estimating variation within the genes and inferring the phylogeny of 186 sequenced diverse Escherichia coli genomes
Source: BMC Genomics. 2012 Oct 31;13:577. doi: 10.1186/1471-2164-13-577 (PMC3575317; doi:10.1186/1471-2164-13-577)

(A)

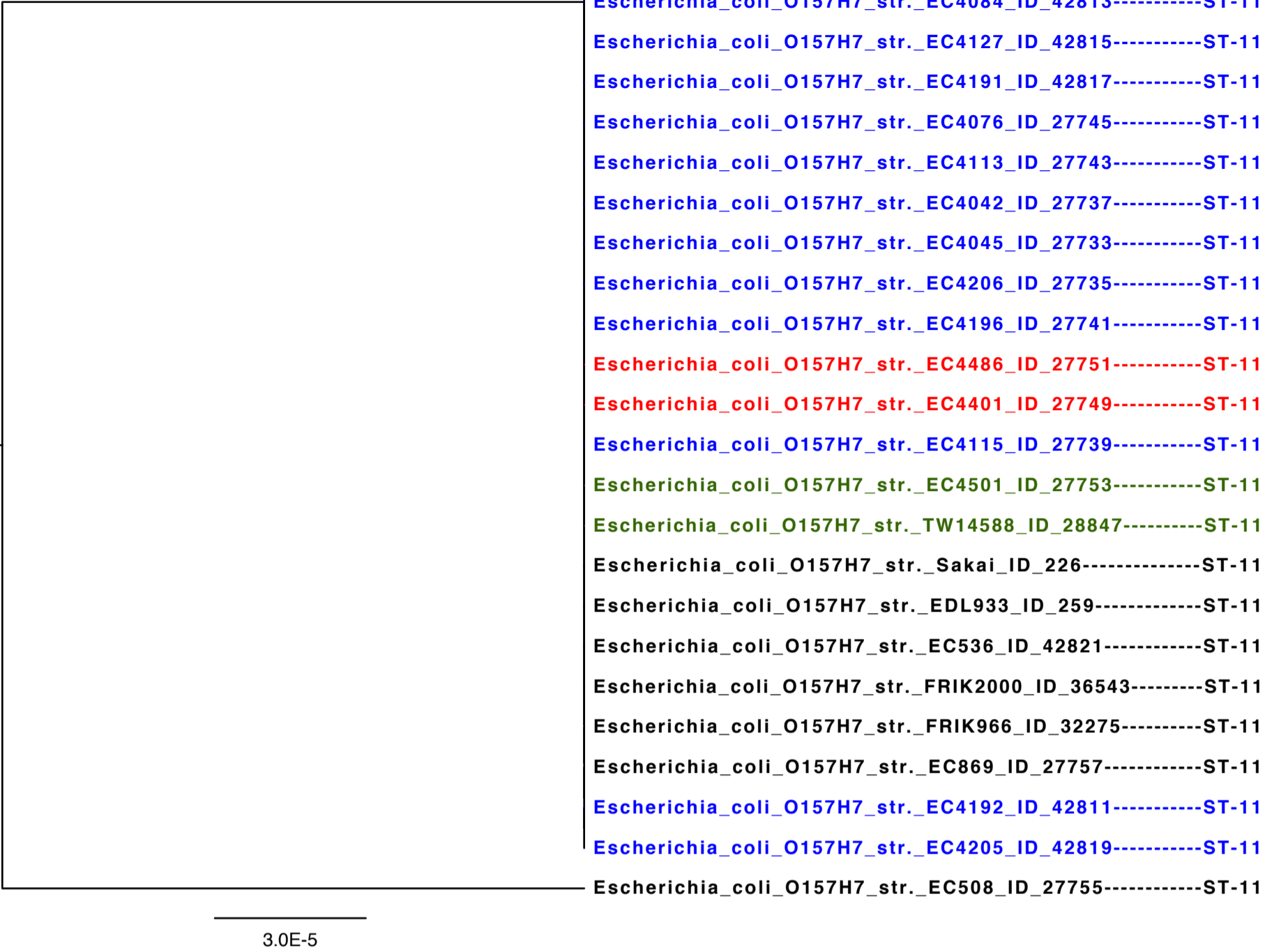

(B)

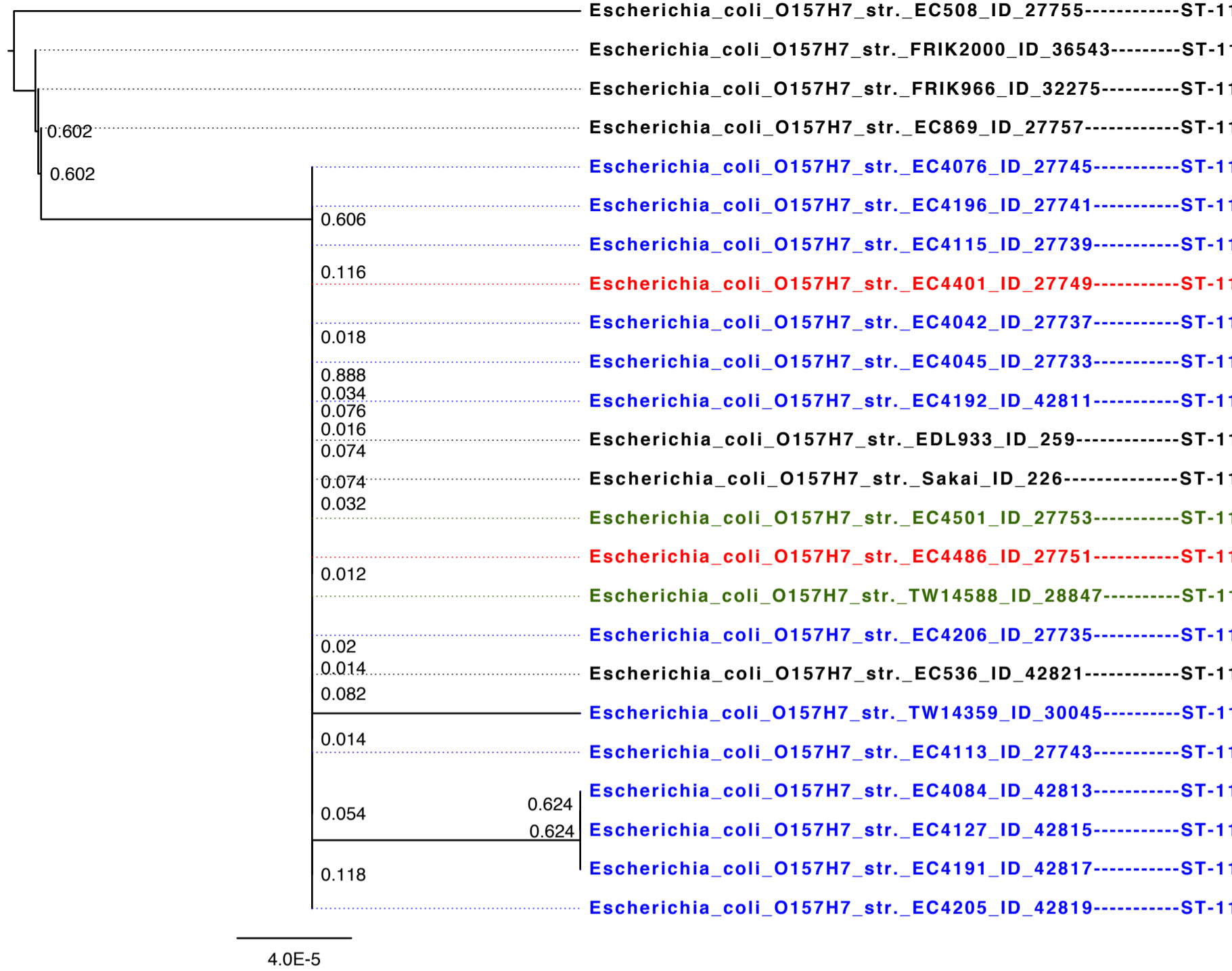

(C)

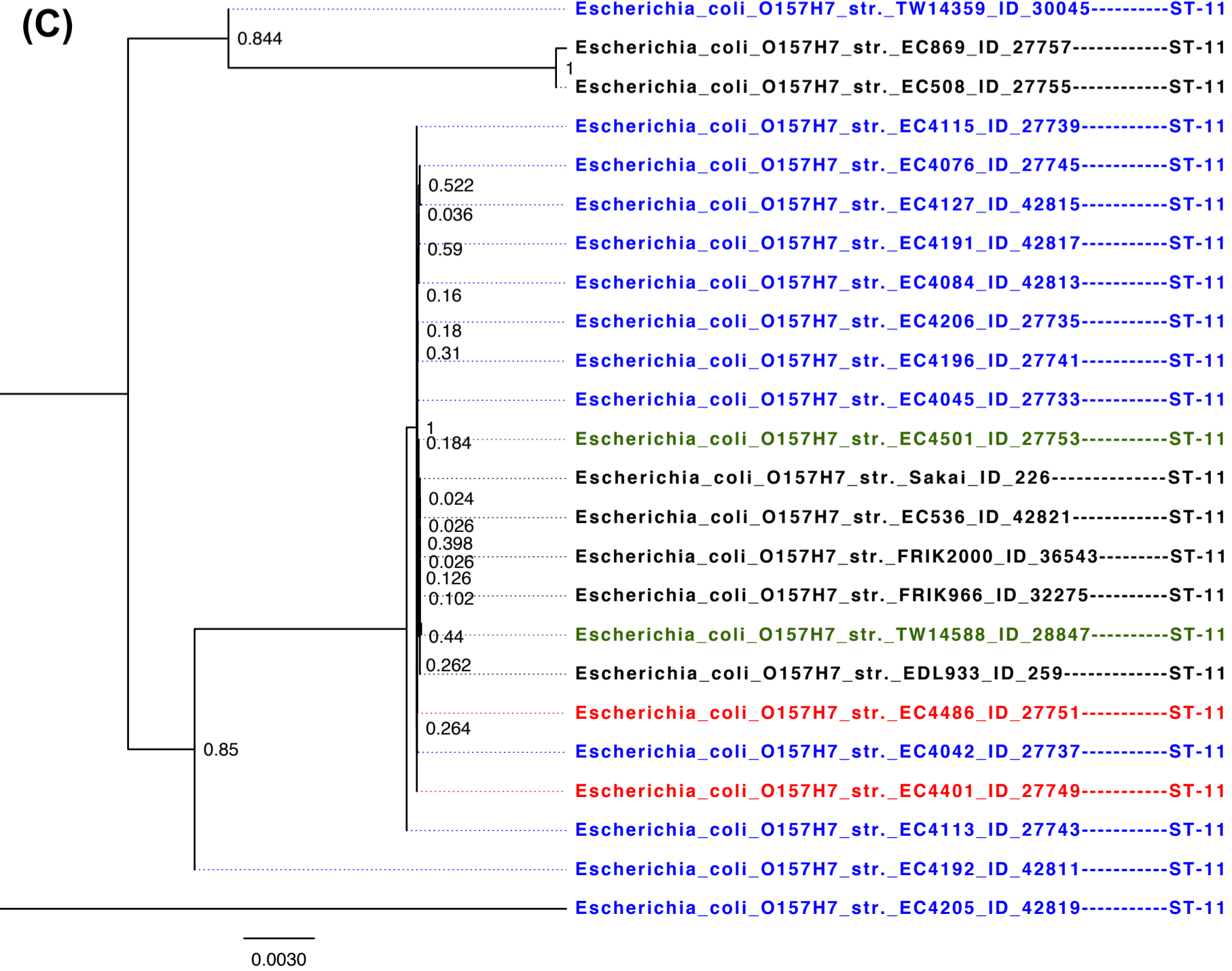

(D)

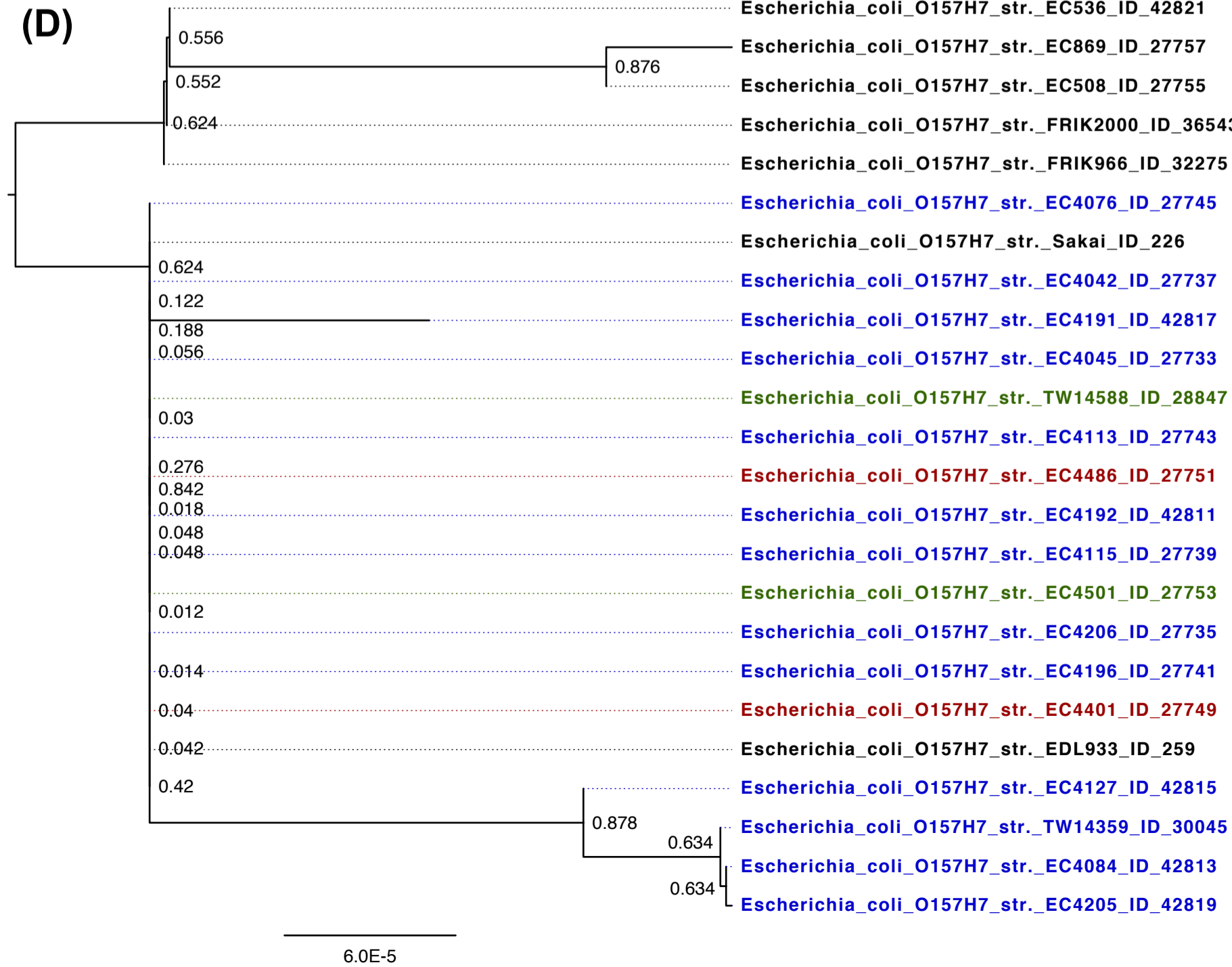

Supplement: Additional file 2 — MLST phylogenies of O157:H7. Four phylogenetic trees inferred from four different MLST schemes. Tree A is inferred from Mark Achtman’s MLST scheme, tree B is inferred from the Pasteur MLST scheme, tree C is inferred from T. Whittam’s MLST scheme and tree D is inferred from the alternative MLST scheme used in this proof of concept case. [file 1471-2164-13-577-S2.pdf]

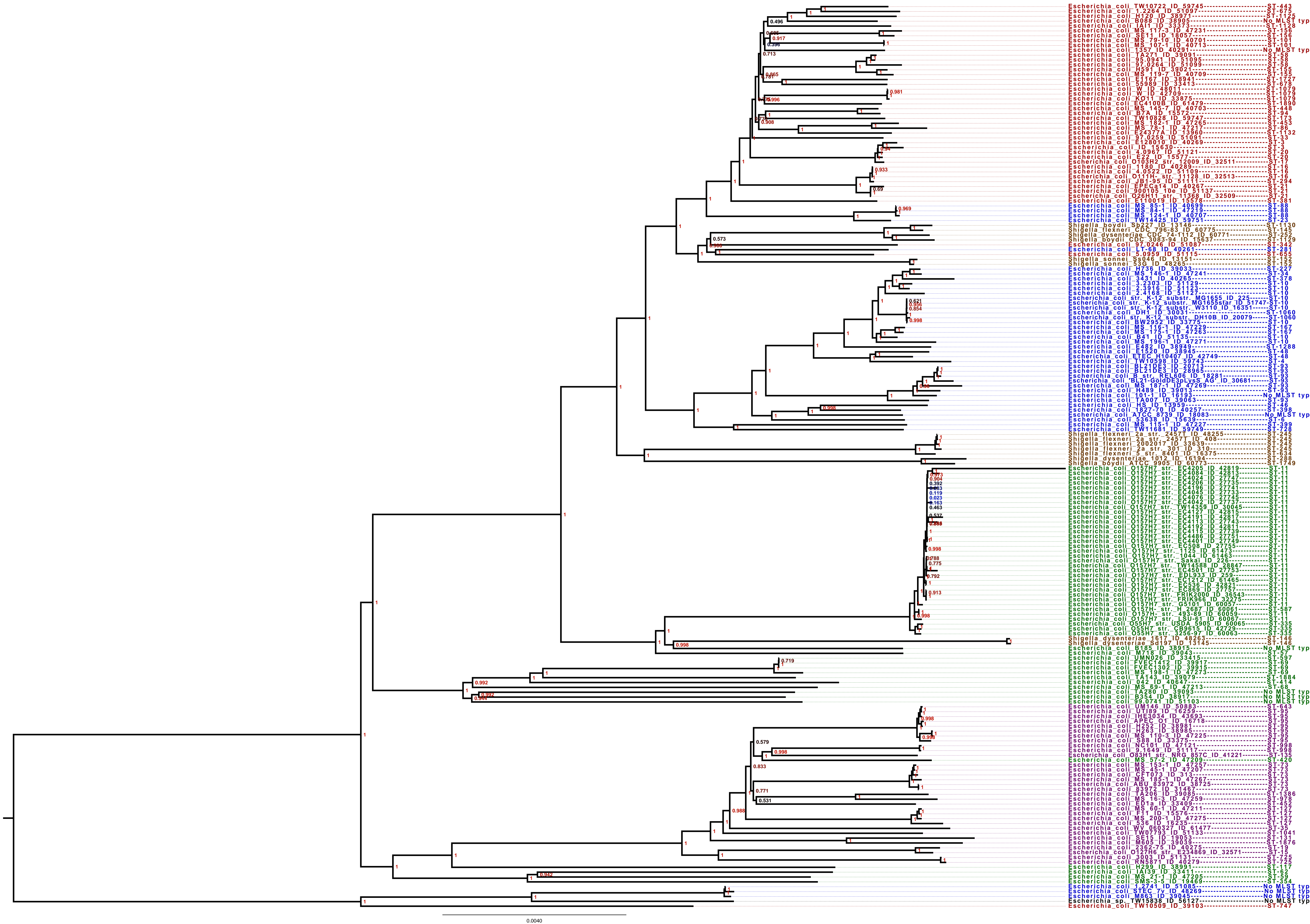

Supplement: Additional file 3 — Core tree with all bootstrap values. The tree was created from the alignment of each of the 1,278 core genes from the 186 E. coli genomes. MLST types are annotated to the far right of each genome name. The phylotypes are marked with the colors blue (A), red (B1), purple (B2), green (D), and the Shigella genomes are marked with the color brown. [file 1471-2164-13-577-S3.pdf]

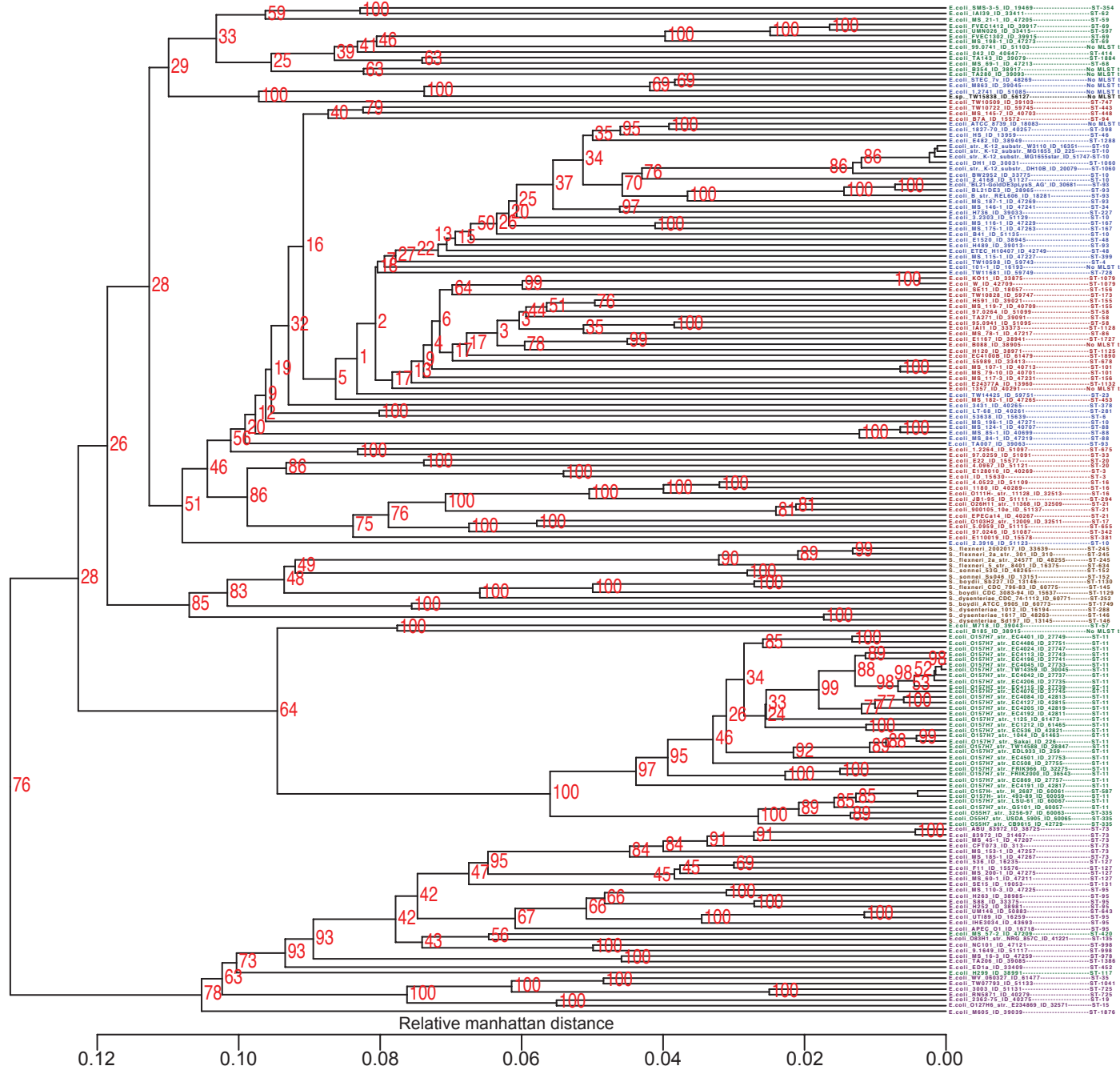

Supplement: Additional file 4 — Pan-genome tree with all bootstrap values. The tree was created based on the presence or absence of 16,373 HGCs in the 186 E. coli genomes. MLST types are annotated to the far right of each genome name. The phylotypes are marked with the colors blue (A), red (B1), purple (B2), green (D), and the Shigella genomes are marked with the color brown. Bootstrap values are annotated at each node as a percentage between 0 and 100. [file 1471-2164-13-577-S4.pdf]

Pan-genome diversity distributions

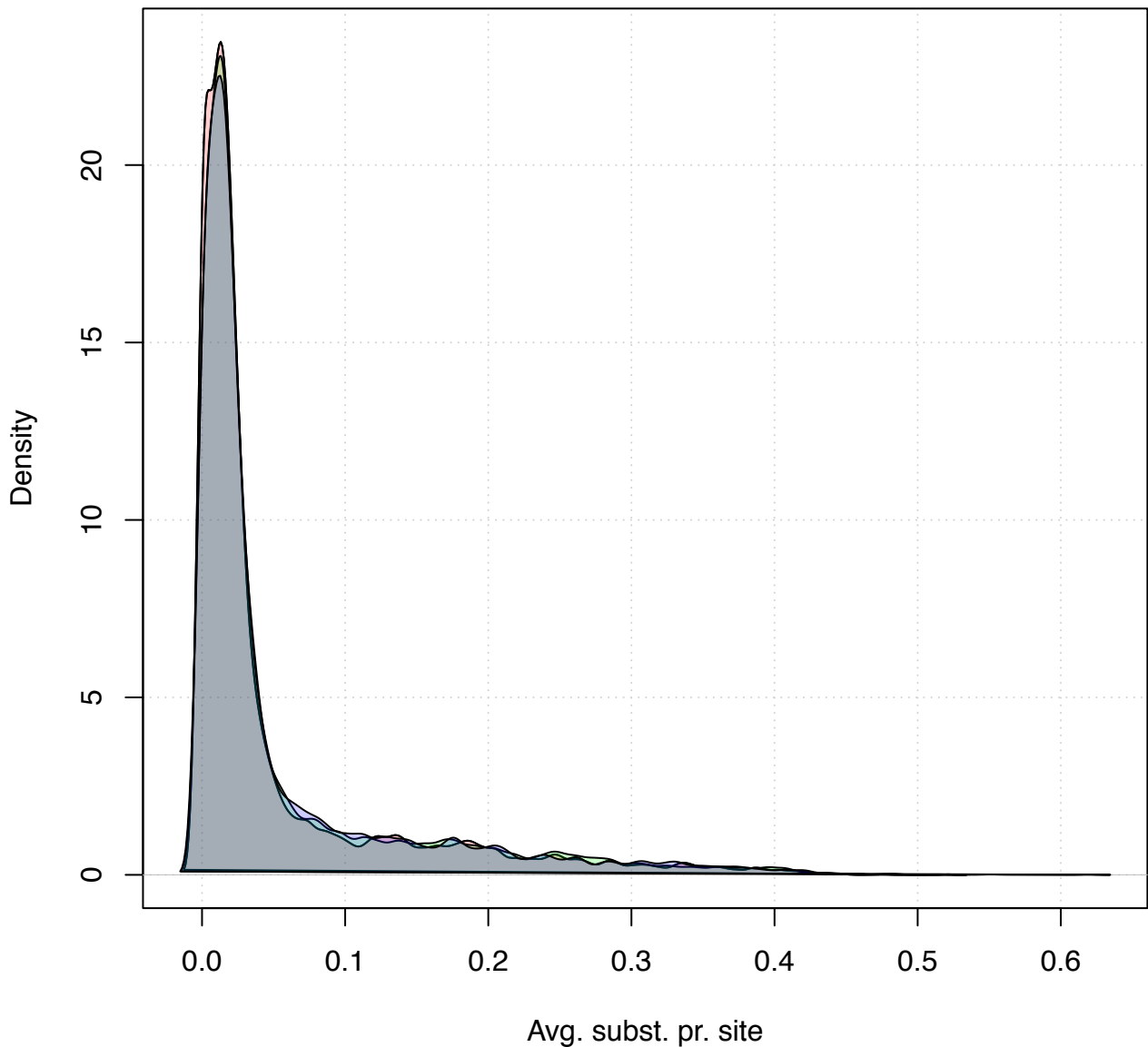

Core-genome diversity distributions

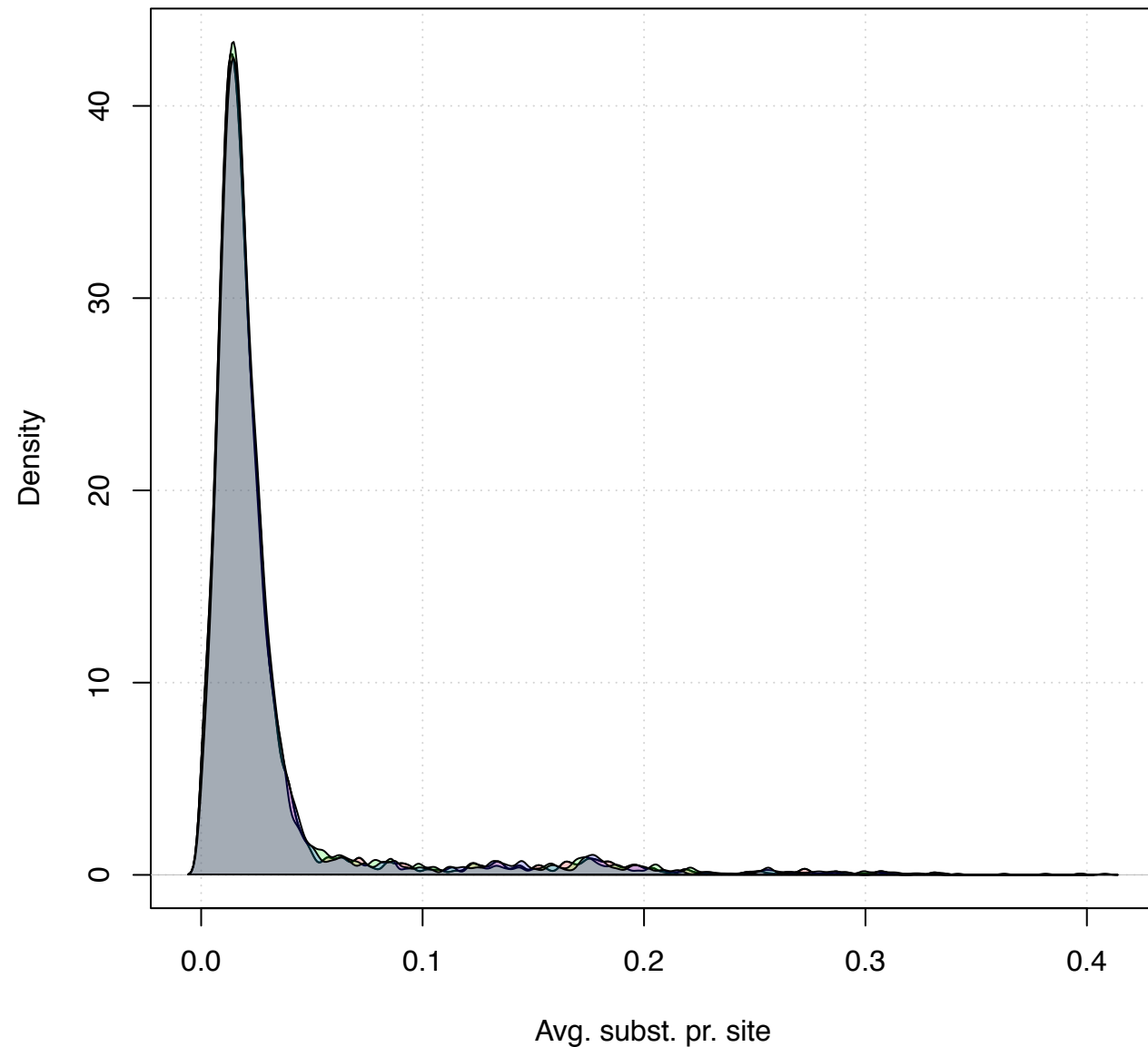

Supplement: Additional file 6 — Complete versus draft nucleotide diversity distributions. The nucleotide diversity distribution is plotted for both the core-HGCs and the pan-HGCs of the three datasets: complete (red), draft1 (blue), and draft2 (green). [file 1471-2164-13-577-S6.pdf]
