# Supplementary material for: Estimating variation within the genes and inferring the phylogeny of 186 sequenced diverse Escherichia coli genomes
Source: BMC Genomics. 2012 Oct 31;13:577. doi: 10.1186/1471-2164-13-577 (PMC3575317; doi:10.1186/1471-2164-13-577)
Supplement: Additional file 5 — Annotation of highly deviating HGCs. Manual annotation of the 10 HGCs with the highest standard deviation in gene size. The annotation is based on blasting the gene members against the nr database, Uniprot and running the sequences through InterProtScan. [file 1471-2164-13-577-S5.pdf]

Std. dev.	Avg. size	Seqs in HGC	Gene / product
4696	13604	53	Adhesin for cattle intestine colonization
3574	5096	20	putative invasin
3468	7125	29	probable hemagglutinin-related protein
3456	5983	47	ToxB (plasmid)
2982	6444	53	Stx1 converting phage
2889	7356	25	Efa1/LifA-like protein
2776	7159	5	putative haemolysin/haemagglutinin
2686	4478	198	Adhesin
2668	4528	9	Efa1/LifA-like protein(Genomic island)
2501	8081	59	yersiniabactin biosynthetic protein
2458	4312	11	defense against restriction protein
2427	6606	56	RatA-like protein
2334	8527	19	putative peptide/polyketide synthase
2298	4047	7	hypothetical protein (K. pneumoniae plasmid)
2105	2842	3	ycbB. Possible Adhesin (plasmid)
2044	2443	4	Adhesin
