# Supplementary material for: Estimating variation within the genes and inferring the phylogeny of 186 sequenced diverse Escherichia coli genomes
Source: BMC Genomics. 2012 Oct 31;13:577. doi: 10.1186/1471-2164-13-577 (PMC3575317; doi:10.1186/1471-2164-13-577)
Supplement: Additional file 7 — Table of complete dataset. The table shows the dataset used for the article. The “GB genes” column indicates the number of genes annotated in the corresponding GenBank file. The “Prod genes” column indicates the number of genes that was found with prodigal for this study. [file 1471-2164-13-577-S7.pdf]

GPID	Name	Status	Contigs	Ns	Plasmids	GB genes	Prodigal genes	16S rRNA	Length	AT content	tRNA	MLST
30681	Escherichia coli 'BL21-Gold(DE3)pLysS AG'	complete	1	0	0	4228	4299	7	4570938	49,16	85	ST-93
40647	Escherichia coli 042	complete	1	0	1	5038	5054	7	5355323	49,46	92	ST-414
16235	Escherichia coli 536	complete	1	0	0	4685	4543	7	4938920	49,48	80	ST-127
33413	Escherichia coli 55989	complete	1	0	0	4919	4845	7	5154862	49,34	93	ST-678
38725	Escherichia coli ABU 83972	complete	1	1	1	4796	4741	7	5132961	49,42	88	ST-73
16718	Escherichia coli APEC O1	complete	1	1	2	4890	5259	7	5497653	49,66	95	ST-95
18083	Escherichia coli ATCC 8739	complete	1	0	0	4200	4393	7	4746218	49,13	86	Unknown
18281	Escherichia coli B str. REL606	complete	1	0	0	4209	4321	7	4629812	49,23	85	ST-93
20713	Escherichia coli BL21(DE3)	complete	1	0	0	4159	4272	7	4558953	49,17	85	ST-93
28965	Escherichia coli BL21(DE3)	complete	1	0	0	4319	4272	7	4558947	49,17	85	ST-93
33775	Escherichia coli BW2952	complete	1	0	0	4084	4280	7	4578159	49,21	86	ST-10
313	Escherichia coli CFT073	complete	1	87	0	5379	4902	7	5231428	49,52	88	ST-73
30031	Escherichia coli DH1	complete	1	0	0	4160	4309	7	4630707	49,19	87	ST-1060
13960	Escherichia coli E24377A	complete	1	0	6	4989	5005	7	5246055	49,44	88	ST-1132
33409	Escherichia coli ED1a	complete	1	100	0	5123	4987	7	5209548	49,27	89	ST-452
42749	Escherichia coli ETEC H10407	complete	1	0	0	4763	4924	7	5153435	49,24	89	ST-48
13959	Escherichia coli HS	complete	1	0	0	4384	4323	7	4643538	49,18	87	ST-46
33373	Escherichia coli IAI1	complete	1	0	0	4443	4328	7	4700560	49,21	85	ST-1128
33411	Escherichia coli IAI39	complete	1	0	0	4892	4862	7	5132068	49,37	87	ST-62
43693	Escherichia coli IHE3034	complete	1	0	0	4757	4816	7	5108383	49,30	96	ST-95
33875	Escherichia coli KO11	complete	1	0	2	4653	4722	7	5029323	49,19	85	ST-1079
32511	Escherichia coli O103:H2 str. 12009	complete	1	0	1	5354	5338	7	5524860	49,37	97	ST-17
32513	Escherichia coli O111:H- str. 11128	complete	1	0	5	5732	5690	7	5766081	49,56	110	ST-16
32571	Escherichia coli O127:H6 str. E2348/69	complete	1	0	2	4824	4873	7	5069678	49,48	90	ST-15
27739	Escherichia coli O157:H7 str. EC4115	complete	1	0	2	5477	5548	7	5704171	49,59	108	ST-11
259	Escherichia coli O157:H7 str. EDL933	complete	1	4228	1	5449	5420	7	5620522	49,54	99	ST-11*
226	Escherichia coli O157:H7 str. Sakai	complete	1	0	2	5447	5398	7	5594477	49,52	102	ST-11
30045	Escherichia coli O157:H7 str. TW14359	complete	1	0	1	5373	5444	7	5622737	49,54	107	ST-11
32509	Escherichia coli O26:H11 str. 11368	complete	1	0	4	5795	5771	7	5855531	49,35	100	ST-21
42729	Escherichia coli O55:H7 str. CB9615	complete	1	0	1	5121	5151	7	5452353	49,50	99	ST-335

41221	Escherichia coli O83:H1 str. NRG 857C	complete	1	0	1	4582	4542	7	4894879	49,31	83	ST-135
33375	Escherichia coli S88	complete	1	0	1	4991	4887	7	5166121	49,35	90	ST-95
18057	Escherichia coli SE11	complete	1	0	6	5002	4895	7	5155626	49,26	87	ST-156
19053	Escherichia coli SE15	complete	1	0	1	4488	4448	7	4839683	49,25	85	ST-131
19469	Escherichia coli SMS-3-5	complete	1	0	4	4913	4822	7	5215377	49,51	89	ST-354
20079	Escherichia coli str. K-12 substr. DH10B	complete	1	0	0	4200	4398	7	4686137	49,22	87	ST-1060
225	Escherichia coli str. K-12 substr. MG1655	complete	1	0	0	4319	4313	7	4639675	49,21	87	ST-10
16351	Escherichia coli str. K-12 substr. W3110	complete	1	0	0	4337	4326	7	4646332	49,20	87	ST-10
50883	Escherichia coli UM146	complete	1	8	1	4783	4804	7	5107563	49,40	84	ST-643
33415	Escherichia coli UMN026	complete	1	53	2	5107	5021	7	5358200	49,34	87	ST-597
16259	Escherichia coli UTI89	complete	1	0	1	5211	4839	7	5179971	49,39	88	ST-95
48011	Escherichia coli W	complete	1	1	2	4700	4705	7	5008864	49,18	87	ST-1079
15637	Shigella boydii CDC 3083-94	complete	1	0	5	4557	5121	7	4874659	48,97	98	ST-1129
13146	Shigella boydii Sb227	complete	1	0	1	4290	4822	7	4646520	48,89	91	ST-1130
13145	Shigella dysenteriae Sd197	complete	1	0	2	4508	5217	7	4560911	49,03	84	ST-146
33639	Shigella flexneri 2002017	complete	1	1	5	4706	5091	7	4894492	49,39	102	ST-245
408	Shigella flexneri 2a str. 2457T	complete	1	1	0	4073	4720	7	4599354	49,09	100	ST-245
310	Shigella flexneri 2a str. 301	complete	1	6	1	4705	5006	7	4828821	49,35	97	ST-245
16375	Shigella flexneri 5 str. 8401	complete	1	0	0	4116	4680	7	4574284	49,08	97	ST-634
13151	Shigella sonnei Ss046	complete	1	0	4	4476	5105	7	5055316	49,23	97	ST-152
15630	Escherichia coli	draft	262	0	1	4626	5579	0	5495385	49,56	75	ST-3
51097	Escherichia coli 1.2264	draft	86	8	0	0	5362	6	5498865	49,43	89	ST-675
51085	Escherichia coli 1.2741	draft	68	4	0	0	5532	7	5657864	49,71	100	Unknown*
16193	Escherichia coli 101-1	draft	91	0	0	4205	4867	2	4979767	49,37	73	Unknown
40289	Escherichia coli 1180	draft	86	1	0	5772	5564	8	5554557	49,50	111	ST-16
40291	Escherichia coli 1357	draft	180	2	0	5230	5137	7	5277070	49,39	86	Unknown
40257	Escherichia coli 1827-70	draft	35	1	0	4707	4654	8	4803089	49,21	88	ST-398
51123	Escherichia coli 2.3916	draft	207	8	0	0	5758	9	5602529	49,36	103	ST-10
51127	Escherichia coli 2.4168	draft	27	3	0	0	4579	7	4757936	49,07	81	ST-10
40275	Escherichia coli 2362-75	draft	83	0	0	5220	5027	8	5173444	49,52	96	ST-19
51129	Escherichia coli 3.2303	draft	51	3	0	0	4810	7	4950291	49,54	91	ST-10

51131	Escherichia coli 3003	draft	21	0	0	0	4709	8	4927221	49,32	90	ST-725
40265	Escherichia coli 3431	draft	127	3	0	5092	5189	6	5223419	49,07	89	ST-378
51109	Escherichia coli 4.0522	draft	125	6	0	0	5920	7	5830788	49,52	118	ST-16
51121	Escherichia coli 4.0967	draft	96	2	0	0	5958	7	5860313	49,66	94	ST-20
51115	Escherichia coli 5.0959	draft	79	6	0	0	5309	6	5370927	49,47	98	ST-655
15639	Escherichia coli 53638	draft	2	0	2	376	5287	7	5371790	49,01	102	ST-6
31467	Escherichia coli 83972	draft	159	6	0	5255	4803	1	5066014	49,43	72	ST-73
51117	Escherichia coli 9.1649	draft	33	0	0	0	4876	8	5102049	49,36	87	ST-998
51137	Escherichia coli 900105 (10e)	draft	63	4	0	0	5552	7	5556164	49,41	102	ST-21
51095	Escherichia coli 95.0941	draft	47	0	0	0	4486	8	4786951	49,13	90	ST-58
51087	Escherichia coli 97.0246	draft	94	5	0	0	5561	11	5475907	49,40	107	ST-342
51091	Escherichia coli 97.0259	draft	76	4	0	0	5500	5	5445504	49,33	88	ST-33
51099	Escherichia coli 97.0264	draft	133	5	0	0	5048	5	5230651	49,38	87	ST-58
51103	Escherichia coli 99.0741	draft	40	5	0	0	4899	7	5209988	49,48	88	Unknown*
38905	Escherichia coli B088	draft	148	4	0	0	4715	1	4944201	49,48	77	Unknown
38915	Escherichia coli B185	draft	115	2	0	0	4755	1	5106858	49,40	83	Unknown*
38917	Escherichia coli B354	draft	69	5	0	0	4474	1	4831934	49,45	76	Unknown*
51135	Escherichia coli B41	draft	34	4	0	0	4843	8	5032803	49,28	87	ST-10
15572	Escherichia coli B7A	draft	289	0	0	4404	5277	2	5300242	49,31	64	ST-94
15578	Escherichia coli E110019	draft	137	0	0	4593	5323	4	5376211	49,28	69	ST-381
38941	Escherichia coli E1167	draft	87	9	0	4597	4706	1	4918295	49,28	70	ST-1727
40269	Escherichia coli E128010	draft	284	1	0	5385	5403	7	5221267	49,41	89	ST-3
38945	Escherichia coli E1520	draft	127	5	0	4613	4749	1	4896881	49,37	79	ST-48
15577	Escherichia coli E22	draft	127	0	0	4828	5418	6	5528238	49,37	75	ST-20
38949	Escherichia coli E482	draft	94	3	0	4528	4660	1	4833169	49,30	72	ST-1288
61479	Escherichia coli EC4100B	draft	42	19	0	4910	4971	3	5111899	49,49	87	ST-1890
40267	Escherichia coli EPECa14	draft	199	5	0	5844	5590	5	5435297	49,54	99	ST-21
15576	Escherichia coli F11	draft	119	0	0	4318	4925	7	5215961	49,51	73	ST-127
39915	Escherichia coli FVEC1302	draft	149	5	0	0	5155	1	5291786	49,37	73	ST-69
39917	Escherichia coli FVEC1412	draft	137	4	0	0	5021	1	5194603	49,30	70	ST-69
38971	Escherichia coli H120	draft	172	2	0	4866	4981	1	5044811	49,53	61	ST-1125

38981	Escherichia coli H252	draft	112	7	0	4996	5098	1	5260709	49,53	84	ST-95
38985	Escherichia coli H263	draft	165	18	0	5011	5008	1	5192679	49,50	78	ST-95
38991	Escherichia coli H299	draft	229	2	0	0	5098	1	5248906	49,25	85	ST-117
39013	Escherichia coli H489	draft	141	3	0	4622	4680	1	4832506	49,09	84	ST-93
39021	Escherichia coli H591	draft	190	9	0	0	4811	1	4918687	49,28	71	ST-155
39033	Escherichia coli H736	draft	135	6	0	0	4445	1	4652606	49,18	71	ST-227
51111	Escherichia coli JB1-95	draft	162	31	0	0	5497	6	5397192	49,52	95	ST-294
40261	Escherichia coli LT-68	draft	63	0	0	5567	5383	8	5189427	49,15	104	ST-281*
39039	Escherichia coli M605	draft	162	3	0	0	5340	1	5446689	49,57	75	ST-1876
39043	Escherichia coli M718	draft	188	6	0	0	5127	1	5365946	49,43	74	ST-57
39045	Escherichia coli M863	draft	152	6	0	4953	5055	1	5250401	49,76	76	Unknown*
40713	Escherichia coli MS 107-1	draft	72	0	0	4625	4638	3	4905150	49,41	80	ST-101
47225	Escherichia coli MS 110-3	draft	411	0	0	5415	4841	1	5063123	49,50	64	ST-95
47227	Escherichia coli MS 115-1	draft	423	0	0	5020	4594	1	4797820	49,24	63	ST-399
47229	Escherichia coli MS 116-1	draft	484	0	0	5158	4621	1	4840256	49,45	61	ST-167
47231	Escherichia coli MS 117-3	draft	467	0	0	5371	4900	0	5013840	49,47	65	ST-156
40709	Escherichia coli MS 119-7	draft	130	0	0	5071	4814	2	4968686	49,25	86	ST-155
40707	Escherichia coli MS 124-1	draft	176	0	0	5668	5293	1	5394127	49,41	77	ST-88
40703	Escherichia coli MS 145-7	draft	143	0	0	5124	4978	1	5115051	49,24	84	ST-448
47241	Escherichia coli MS 146-1	draft	355	0	0	4953	4516	0	4740105	49,27	61	ST-34
47257	Escherichia coli MS 153-1	draft	507	0	0	5465	4878	1	5087744	49,52	64	ST-73
47259	Escherichia coli MS 16-3	draft	451	0	0	5289	4743	1	4952805	49,46	58	ST-978
47263	Escherichia coli MS 175-1	draft	413	0	0	4866	4429	1	4673288	49,30	63	ST-167
47265	Escherichia coli MS 182-1	draft	456	0	0	5315	4849	1	4982225	49,46	67	ST-453
47267	Escherichia coli MS 185-1	draft	378	0	0	5160	4659	0	4939390	49,38	56	ST-73
47269	Escherichia coli MS 187-1	draft	310	0	0	4453	4112	1	4384173	49,18	64	ST-93
47271	Escherichia coli MS 196-1	draft	785	0	0	5586	5233	1	5214638	49,34	70	ST-10
47273	Escherichia coli MS 198-1	draft	564	0	0	5604	5105	1	5236490	49,33	64	ST-69
47275	Escherichia coli MS 200-1	draft	445	0	0	5342	4765	0	5042708	49,51	56	ST-127
47205	Escherichia coli MS 21-1	draft	540	0	0	5742	5259	1	5282923	49,61	67	ST-59
47207	Escherichia coli MS 45-1	draft	396	0	0	5233	4702	1	4994869	49,59	62	ST-73

47209	Escherichia coli MS 57-2	draft	408	0	0	5225	4767	1	4938310	49,30	63	ST-420*
47211	Escherichia coli MS 60-1	draft	464	0	0	5645	5025	0	5196275	49,49	65	ST-127
47213	Escherichia coli MS 69-1	draft	425	0	0	5426	4952	1	5207549	49,62	63	ST-68*
47217	Escherichia coli MS 78-1	draft	410	0	0	4990	4536	1	4752681	49,37	60	ST-86
40701	Escherichia coli MS 79-10	draft	157	1	0	0	4719	1	4900086	49,41	75	ST-101
47219	Escherichia coli MS 84-1	draft	432	0	0	5709	5109	1	5287926	49,45	71	ST-88
40699	Escherichia coli MS 85-1	draft	234	0	0	5537	5345	1	5457657	49,38	85	ST-88
47121	Escherichia coli NC101	draft	27	0	0	4698	4660	1	5021144	49,43	71	ST-998
60059	Escherichia coli O157:H- str. 493-89	draft	204	0	0	4692	4864	1	5054824	49,47	82	ST-11
60061	Escherichia coli O157:H- str. H 2687	draft	209	0	0	4631	4864	1	5048194	49,46	83	ST-587
61463	Escherichia coli O157:H7 str. 1044	draft	70	1	0	5352	5329	2	5487708	49,57	90	ST-11
61473	Escherichia coli O157:H7 str. 1125	draft	57	1	0	5449	5393	3	5569621	49,68	99	ST-11
61465	Escherichia coli O157:H7 str. EC1212	draft	32	1	0	4914	5302	7	5510053	49,58	102	ST-11
42809	Escherichia coli O157:H7 str. EC4009	draft	277	0	0	0	5076	1	5194891	49,78	58	ST-11
27747	Escherichia coli O157:H7 str. EC4024	draft	364	0	0	0	6332	3	6199307	49,26	103	ST-11
27737	Escherichia coli O157:H7 str. EC4042	draft	3	0	1	1398	5488	7	5617728	49,54	107	ST-11
27733	Escherichia coli O157:H7 str. EC4045	draft	7	0	1	2548	5467	7	5634850	49,53	107	ST-11
27745	Escherichia coli O157:H7 str. EC4076	draft	135	0	0	4745	5613	10	5705645	49,44	109	ST-11
42813	Escherichia coli O157:H7 str. EC4084	draft	881	0	0	0	5705	1	5298036	49,78	68	ST-11
27743	Escherichia coli O157:H7 str. EC4113	draft	231	0	0	4735	5705	5	5655847	49,40	89	ST-11
42815	Escherichia coli O157:H7 str. EC4127	draft	791	0	0	0	5527	1	5296992	49,77	62	ST-11
42817	Escherichia coli O157:H7 str. EC4191	draft	267	0	0	0	5067	1	5201104	49,78	50	ST-11
42811	Escherichia coli O157:H7 str. EC4192	draft	958	0	0	0	5684	1	5349693	49,76	66	ST-11*
27741	Escherichia coli O157:H7 str. EC4196	draft	186	0	0	4596	5565	8	5620606	49,45	95	ST-11
42819	Escherichia coli O157:H7 str. EC4205	draft	1583	0	0	0	6490	1	5287055	49,79	68	ST-11
27735	Escherichia coli O157:H7 str. EC4206	draft	6	1	1	1084	5555	7	5629932	49,53	106	ST-11
27749	Escherichia coli O157:H7 str. EC4401	draft	186	0	0	4581	5701	8	5733133	49,54	95	ST-11
27751	Escherichia coli O157:H7 str. EC4486	draft	165	0	0	4954	5872	8	5933166	49,58	105	ST-11
27753	Escherichia coli O157:H7 str. EC4501	draft	250	0	0	4626	5715	5	5677181	49,42	74	ST-11
27755	Escherichia coli O157:H7 str. EC508	draft	272	0	0	4701	5640	7	5656666	49,35	82	ST-11
42821	Escherichia coli O157:H7 str. EC536	draft	678	0	0	0	5398	1	5262452	49,76	70	ST-11

27757	Escherichia coli O157:H7 str. EC869	draft	147	0	0	4761	5648	8	5731065	49,46	93	ST-11
36543	Escherichia coli O157:H7 str. FRIK2000	draft	247	5	0	0	5325	1	5408690	49,82	77	ST-11
32275	Escherichia coli O157:H7 str. FRIK966	draft	316	8	0	0	5343	1	5376914	49,76	76	ST-11
60057	Escherichia coli O157:H7 str. G5101	draft	216	0	0	4679	4780	1	4971794	49,36	85	ST-11
60067	Escherichia coli O157:H7 str. LSU-61	draft	189	0	0	4627	4820	1	5048276	49,39	86	ST-11
28847	Escherichia coli O157:H7 str. TW14588	draft	10	902	1	8972	10944	14	11249113	49,51	200	ST-11
60063	Escherichia coli O55:H7 str. 3256-97	draft	188	0	0	4684	4865	1	5083624	49,43	87	ST-335
60065	Escherichia coli O55:H7 str. USDA 5905	draft	154	0	0	4669	4877	1	5105283	49,51	88	ST-335
41499	Escherichia coli OP50	draft	2939	0	0	0	6135	0	4418649	49,23	16	Unknown*
40279	Escherichia coli RN587/1	draft	73	1	0	4935	4919	7	5061577	49,39	85	ST-725
48269	Escherichia coli STEC_7v	draft	29	1	0	5072	4875	8	5195834	49,59	103	Unknown*
51747	Escherichia coli str. K-12 substr. MG1655star	draft	52	0	0	0	4339	1	4607632	49,07	87	ST-10
39063	Escherichia coli TA007	draft	232	8	0	5065	5277	1	5252119	49,50	70	ST-93*
39079	Escherichia coli TA143	draft	168	4	0	0	4599	1	4829725	49,42	69	ST-1884
39085	Escherichia coli TA206	draft	187	4	0	0	4902	1	5055289	49,47	62	ST-1386
39091	Escherichia coli TA271	draft	191	7	0	0	4934	1	5020687	49,28	64	ST-58
39093	Escherichia coli TA280	draft	168	5	0	0	5210	1	5258161	49,41	77	Unknown*
51133	Escherichia coli TW07793	draft	35	8	0	0	4383	6	4650895	49,33	84	ST-1041
39103	Escherichia coli TW10509	draft	92	1	0	4878	5099	1	5353500	49,66	82	ST-747
59743	Escherichia coli TW10598	draft	310	2	0	0	5269	4	5243318	49,39	89	ST-4
59745	Escherichia coli TW10722	draft	405	11	0	0	5838	9	5689893	49,46	102	ST-443
59747	Escherichia coli TW10828	draft	227	6	0	0	5181	5	5280267	49,36	90	ST-173
59749	Escherichia coli TW11681	draft	153	5	0	0	5186	8	5305843	49,31	92	ST-728*
59751	Escherichia coli TW14425	draft	155	1	0	0	5171	8	5210696	49,41	90	ST-23
42709	Escherichia coli W	draft	88	250	0	4268	4716	1	4941083	49,22	68	ST-1079
61477	Escherichia coli WV_060327	draft	28	0	0	4434	4424	3	4681497	49,38	82	ST-35*
60773	Shigella boydii ATCC 9905	draft	110	0	0	5125	5379	2	5129830	49,34	84	ST-1749
16194	Shigella dysenteriae 1012	draft	189	0	0	4019	5499	3	5235535	49,44	72	ST-288
48263	Shigella dysenteriae 1617	draft	67	0	0	5833	5348	7	4613557	48,81	85	ST-146
60771	Shigella dysenteriae CDC 74-1112	draft	242	13	0	4644	4921	1	4596141	48,92	80	ST-252
48255	Shigella flexneri 2a str. 2457T	draft	71	4	0	5233	5029	6	4825496	49,26	105	ST-245

60775	Shigella flexneri CDC 796-83	draft	209	1	0	4811	4960	2	4682763	49,10	87	ST-145
48265	Shigella sonnei 53G	draft	150	5	0	5756	5636	7	5185984	49,26	96	ST-152
